# A Phase Recovery Technique Using the Genetic Algorithm for Aberration Correction in a Coherent Imaging System

**DOI:** 10.3390/s23187679

**Published:** 2023-09-05

**Authors:** Yu Zhang, Hongwen Zhang, Guoqin Yuan

**Affiliations:** 1 Changchun Institute of Optics, Fine Mechanics and Physics, Chinese Academy of Sciences, Changchun 130000, China; zhangyuqx@163.com (Y.Z.);; 2 University of Chinese Academy of Sciences, Beijing 100000, China

**Keywords:** image quality, phase recovery, computational imaging, coherent imaging

## Abstract

For traditional imaging systems, high imaging quality and system miniaturization are often contradictory. In order to meet the requirements of high imaging quality and system miniaturization, this paper proposes a method to correct the aberration of coherent imaging optical systems. The method is based on the idea of phase recovery and the imaging principle of a coherent imaging system to recover the aberrations at the exit pupil of the system. According to the recovered aberrations, conjugate filters are constructed to correct the image quality in the frequency domain. The imaging quality of the system is improved without changing the original optical path, and the simplicity of the system is guaranteed. To solve the pupil frequency domain aberration more accurately, this paper adopts the dual competition and parallel recombination strategy based on the genetic algorithm and introduces the disaster model. The improved genetic algorithm can effectively restrain the appearance of the “precocity” phenomenon. Finally, the paraxial imaging optical path is simulated and verified by experiments. The results show that, after aberration correction, the image sharpness is improved and the edge information is richer, which verifies the feasibility of the coherent imaging system image quality enhancement method proposed in this paper.

## 1. Introduction

In 1972, to solve the imaging problem of electron microscopy, Gerchberg and Saxton et al. proposed a method to recover the phase of objects through iteration [1], namely, the GS iterative phase recovery algorithm (GS algorithm). The core idea of the GS algorithm is an inverse solution, which calculates the phase information that is difficult to obtain through the intensity information that is easy to obtain. After the research and improvement of many researchers, such as Misell and Fienup [2,3,4], this algorithm can extract the phase information of objects at the pixel level with high recovery accuracy and is not affected by the shape of objects. Currently, it is widely used in various fields, such as X-ray crystallography, electron microscopy, wavefront detection, astronomy, and holographic technology [5,6,7,8].

The coherent imaging system is a kind of imaging system that requires high coherence of the light source. When the coherence of time and space is poor, the clarity and visibility of the diffraction pattern will be greatly reduced. With the development of the phase recovery algorithm, more and more researchers focus on improving the image quality of coherent imaging systems by reconstructing pupil function. In 2021, Yao et al. [9], of the Zhejiang University, obtained the PSF (point diffusion function) of each sub-aperture of the system through wavefront coding, then reconstructed pupil function using a phase recovery algorithm, then obtained system PSF based on reconstructed pupil function, and then improved the image quality of the system through deconvolution. In 2022, Wang et al. [10], of the Foshan University, improved the quality of reconstructed images by optimizing spectral function and pupil function based on a phase recovery strategy.

In this paper, a method to improve the image quality of coherent imaging optical systems is proposed based on a phase recovery algorithm. This method combines the phase recovery algorithm with the imaging principle of the coherent imaging system, eliminates the surface propagation of the internal optical path, and does not need to obtain the PSF of the system. The frequency domain conjugate aberration filter is obtained by solving the pupil frequency domain aberration, thereby constructing the frequency domain aberration correction model of the coherent imaging system. By improving the imaging quality of the coherent imaging system, the imaging resolution and accuracy can be improved, the noise and error of the system can be reduced, and the stability of the system can be improved.

## 2. Exit Pupil Frequency Domain Aberration Correction Model Based on Frequency Domain Phase Recovery Algorithm

### 2.1. Aberration Correction Model in Exit Pupil Frequency Domain

In the process of optical imaging, the optical system can be simplified as a “black box” with only the entry pupil and exit pupil. Under the condition of a constant halo, as long as the properties of the exit pupil and entry pupil can be determined at both ends, it is unnecessary to consider the internal structure. The propagation of light between the entry pupil and the exit pupil is described by geometric optics, while the diffraction effect of wave optics is observed before and after the entry pupil. For diffractive-constrained systems, the propagation of light is equivalent to passing through a low-pass filter due to the aperture limitation. The image formed by the system is convolved with the geometrically predicted image and the point-spread function, which can be regarded as the Fraunhofer diffraction pattern of the exit pupil function [11].

(1) $$\begin{array}{c} H\left(f_{x},f_{y}\right)=F\left\{\frac{A}{\lambda z}\iint P\left(x,y\right)e^{-i2\pi \left(f_{x}x+f_{y}y\right)}dxdy\right\}\\ \;\;\;\;\;\;\;=\left(A\lambda z\right)P\left(-\lambda zf_{x},-\lambda zf_{y}\right)=\left(A\lambda z\right)P\left(-x,-y\right) \end{array}$$

where z is exit pupil distance, Aλz is a coefficient, and $P\left(x,y\right)$ is pupil function.

As can be seen from Equation (1), the transfer function of the system is equal to a calibrated pupil function. When the exit pupil is circular, the minus sign of the independent variable can be omitted.

(2) $$H\left(f_{x},f_{y}\right)=P\left(x,y\right)=\left\{\begin{array}{l} 1\;\;\mathrm{inside}\;\mathrm{the}\;\mathrm{pupil}\\ 0\;\;\;\;\;\mathrm{outside}\;\mathrm{the}\;\mathrm{pupil} \end{array}\right.,$$

Cut-off frequency fcx=fcy=D2λdi, where D is pupil diameter and $d_{i}$ is image distance.

Equation (2) is for the system with ideal diffraction limitation. In the actual imaging process, aberration will inevitably exist, which is equivalent to adding a phase factor to the pupil function, as shown in Equation (3):

(3) $$H_{a}\left(f_{x},f_{y}\right)=P\left(x,y\right)e^{ikw\left(x,y\right)}=\left\{\begin{array}{l} e^{ikw\left(x,y\right)}\;\;\mathrm{inside}\;\mathrm{the}\;\mathrm{pupil}\\ \;0\;\;\;\;\mathrm{outside}\;\mathrm{the}\;\mathrm{pupil} \end{array}\right.,$$

If the spectrum of the object is $U_{0}\left(f_{x},f_{y}\right)$ and the spectrum of the image is $U_{i}\left(f_{x},f_{y}\right)$, then the imaging process can be expressed as:

(4) $$U_{i}\left(f_{x},f_{y}\right)=U_{0}\left(f_{x},f_{y}\right)H_{a}\left(f_{x},f_{y}\right)=U_{0}\left(f_{x},f_{y}\right)H\left(f_{x},f_{y}\right)e^{ikw\left(x,y\right)},$$

If the aberration at the exit pupil of the system is known, a conjugate aberration filter $H_{ac}=e^{-ikw\left(x,y\right)}$ can be constructed to correct the aberration at the exit pupil. The correction process is as follows:

(5) $$U_{i}^{\prime }\left(f_{x},f_{y}\right)=U_{i}\left(f_{x},f_{y}\right)H_{ac}=U_{0}\left(f_{x},f_{y}\right)H\left(f_{x},f_{y}\right),$$

### 2.2. Solution of Exit Pupil Aberration Based on Phase Recovery

The premise of constructing the filter is to know the exit pupil frequency domain aberration distribution. In order to calculate the exit pupil frequency domain aberration distribution, this paper proposes a method to solve the exit pupil aberration based on a focal plane and a defocus surface.

As can be seen from Equation (3), pupil function $P\left(x,y\right)=e^{ikw\left(x,y\right)}=e^{i\varphi \left(x,y\right)}$, where $\varphi \left(x,y\right)$ is the wave aberration at exit pupil. In order to describe this wave aberration mathematically, this paper adopts the Zernike polynomial to fit the aberration, that is, $\varphi \left(x,y\right)=\sum_{j=1}^{N}a_{j}Z_{j}$, $Z_{j}$ are the Zernike terms, which are used to represent various aberrations and $a_{j}$ is the Zernike coefficient of the corresponding terms. Since the Zernike polynomial is an orthogonal polynomial in the unit circle, each Zernike coefficient can be handled independently in the analysis and correction of aberration and the coefficients will not affect each another [12].

After fitting the aberration by the Zernike polynomial, the solution of the aberration distribution at the exit pupil is to find the value of each coefficient of the Zernike polynomial. Assuming that the amplitude distribution of the Gauss light source is $u_{0}\left(x_{0},y_{0}\right)$, it is converted to the frequency domain $U_{0}\left(f_{x_{0}},f_{y_{0}}\right)$ by Fourier transform. The solution process of the aberration in the frequency domain of the exit pupil is as follows:

1. Use Zernike polynomials to fit aberrations $P\left(x,y\right)=\exp\left(i\sum_{j=1}^{N}a_{j}Z_{j}\right)$. According to the aberration correction model, the transfer function of the system $H_{a}\left(f_{x},f_{y}\right)=P\left(x,y\right)=\exp\left(i\sum_{j=1}^{N}a_{j}Z_{j}\right)$;
2. According to the imaging principle of optical system, the image frequency spectrum is equal to the product of the object frequency spectrum and the transfer function of the system $U_{1}\left(f_{x_{1}},f_{y_{1}}\right)=U_{0}\left(f_{x_{0}},f_{y_{0}}\right)H_{a}\left(f_{x},f_{y}\right)$. The optical field distribution at the focal plane can be obtained by the inverse Fourier transform $u_{1}\left(x_{1},y_{1}\right)=F^{-1}\left\{U_{1}\left(f_{x_{1}},f_{y_{1}}\right)\right\}=\left|u_{1}\left(x_{1},y_{1}\right)\right|e^{i\varphi_{1}\left(x_{1},y_{1}\right)}$. Replace the calculated optical field amplitude distribution with the actual measured focal plane complex amplitude distribution $A_{1}\left(x_{1},y_{1}\right)$, forming a new focal plane optical field distribution $u_{1}^{\prime }\left(x_{1},y_{1}\right)=A_{1}\left(x_{1},y_{1}\right)e^{i\varphi_{1}\left(x_{1},y_{1}\right)}$;
3. The optical field at the focal plane is diffracted to the defocusing plane and the optical field distribution at the defocusing plane is obtained according to the angular frequency spectrum diffraction:

$$\begin{array}{c} \;\;\;\;\;\;\;\;\;u_{2}\left(x_{2},y_{2}\right)=F^{-1}\left\{F\left[u_{1}^{\prime }\left(x_{1},y_{1}\right)\right]\exp\left[ikL_{1}\sqrt{1-{\left(\lambda f_{x_{2}}\right)}^{2}-{\left(\lambda f_{y_{2}}\right)}^{2}}\right]\right\}\\ =\left|u_{2}\left(x_{2},y_{2}\right)\right|e^{i\varphi_{2}\left(x_{2},y_{2}\right)} \end{array}$$

4. Use the diffraction amplitude $\left|u_{2}\left(x_{2},y_{2}\right)\right|$ of the optical field distribution at the defocusing plane and the measured amplitude $A_{2}\left(x_{2},y_{2}\right)$ to establish the evaluation function $E={\sum_{x_{2},y_{2}}\left[\left|u_{2}\left(x,y\right)\right|-A_{2}\left(x_{2},y_{2}\right)\right]}^{2}$;
5. Use the optimization algorithm to optimize the evaluation function, obtain its minimum value, calculate the values of each Zernike coefficient at the minimum value, return to step (1), and conduct cyclic iteration. When the evaluation function is lower than the set threshold or reaches the maximum number of cycles, the iteration stops and the coefficients of each Zernike polynomial are output at this time.

**Figure 1:**
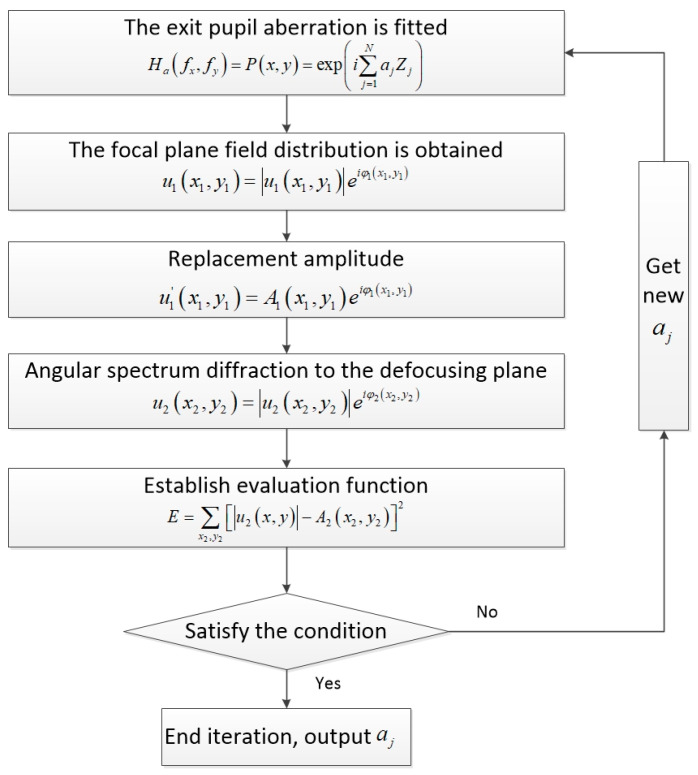
Flow chart of exit pupil phase recovery.

**Figure 2:**
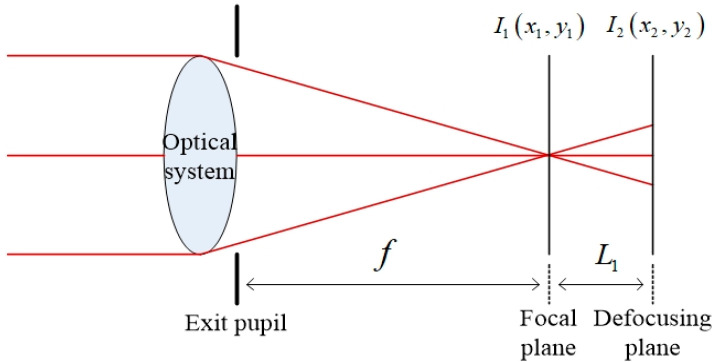
Imaging system diagram.

The flowchart of the entire process is shown in Figure 1:

After the iteration of the above five steps, the value of the Zernike polynomial is obtained and the aberration distribution in the exit pupil frequency domain of the coherent imaging system can be obtained by using this value for aberration fitting. The whole process requires only moving the detector back and forth to obtain the optical field distribution $I_{1}\left(x_{1},y_{1}\right)$ and $I_{2}\left(x_{2},y_{2}\right)$ of the two planes. The amplitude distribution is $A_{1}\left(x_{1},y_{1}\right)=\sqrt{I_{1}\left(x_{1},y_{1}\right)}$ and $A_{2}\left(x_{2},y_{2}\right)=\sqrt{I_{2}\left(x_{2},y_{2}\right)}$. Figure 2 shows the optical path.

## 3. Solving the Aberration in the Exit Pupil Frequency Domain Based on the Genetic Algorithm

According to the analysis, the evaluation function in this paper is relatively complex, and the optimization algorithm based on the gradient is poor in solving such functions. With the development of optimization theory, inspired by the law of natural phenomena and the sociality of biological groups, many intelligent optimization algorithms have been developed [13,14]. The genetic algorithm (GA for short) is an optimal solution search algorithm that simulates the natural evolution process and simulates Darwinian biological evolution theory of “survival of the fittest” [15,16]. The GA has been widely used since it was proposed by Professor John H. Holland in 1975. As a practical, efficient, and robust optimization technique, the genetic algorithm is mainly characterized by direct operation on structural objects, no restriction on the derivation and function continuity, inherent implicit parallelism, and better global optimization ability. In addition, the genetic algorithm adopts a probabilistic optimization method, which can adjust the search direction adaptively without definite rules. Based on the significant advantages of the genetic algorithm, this paper chooses to use genetic algorithm to optimize the evaluation function. And to improve the process of solution, based on the classical genetic algorithm, dual competition and parallel recombination strategy are proposed.

**Figure 3:**
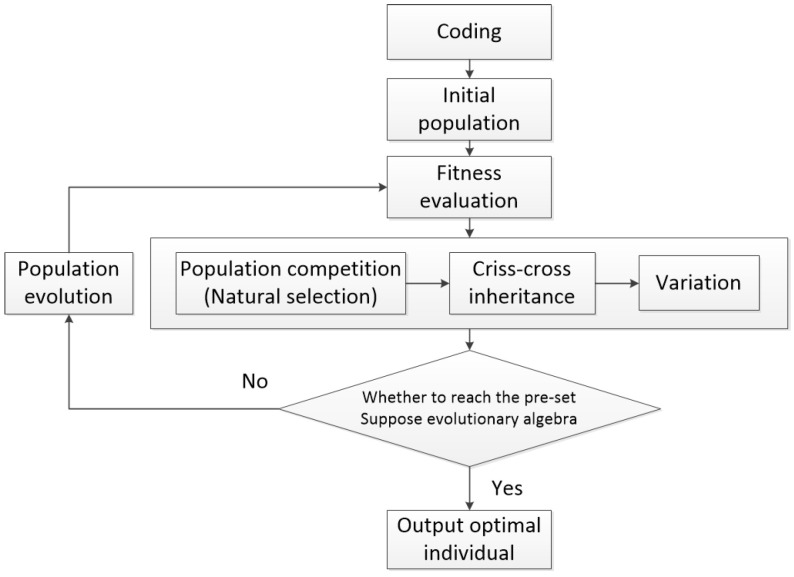
Flow chart of the genetic algorithm principle.

The classical genetic algorithm consists of three basic operations: selection, inheritance, and mutation. It combines individual “heredity and mutation” with nature’s “survival of the fittest” to find the optimal individual. The specific process is shown in the Figure 3.

To verify the performance of the genetic algorithm in solving aberration distribution, the imaging system is simulated in this section. The exit pupil size of the simulated imaging system is 5 mm and the focal length is 50 mm. Due to the use of angular spectral diffraction, the receiving surface size of the image square is also set to 5 mm × 5 mm, and the light source is a monochromatic parallel light source of 632.8 nm. According to $r_{airy}=1.22\lambda \frac{f}{D}$, the radius of the Airy spot is known $r_{airy}=7.74\;\mu m$. In order to make the sampling rate of the detector match it, the simulation sampling number is 1024 × 1024, and the pixel size is 4.88 μm. The wave aberrations in the exit pupil plane were fitted using defocus (*Z*1), astigmatism (*Z*2, *Z*3) (45° and 0/90°), coma (Z4, Z5) (x and y directions), and primary spherical aberration (*Z*6) in Zernike aberration. The normalized Zernike polynomial expression of the six-term aberration is as follows:

(6) $$\begin{array}{l} Z1=\sqrt{3}\left(2x^{2}+2y^{2}-1\right)\\ Z2=\sqrt{6}\left(x^{2}-y^{2}\right)\\ Z3=\sqrt{6}\cdot 2xy\\ Z4=\sqrt{8}\left[-2x+3x\left(x^{2}+y^{2}\right)\right]\\ Z5=\sqrt{8}\left[-2y+3y\left(x^{2}+y^{2}\right)\right]\\ Z6=\sqrt{5}\left(6x^{4}+12x^{2}y^{2}+6y^{4}-6x^{2}-6y^{2}+1\right) \end{array}$$

**Figure 4:**
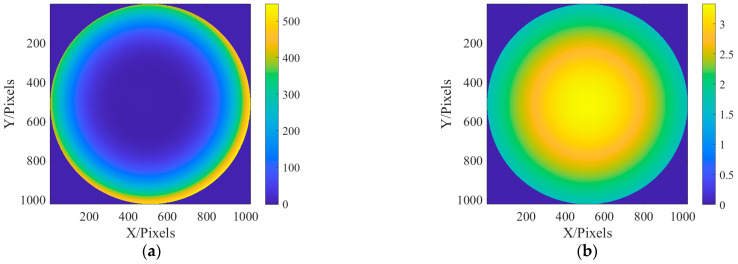
The distribution of exit pupil aberration and light source amplitude are fitted: (**a**) the fitted exit pupil aberration; and (**b**) the amplitude of light source.

Due to the parallel incident light in this paper, there is only on-axis aberration in principle, but there are also some off-axis aberrations in the paraxial field of view. Therefore, in the simulation, the aberrations on the defocus and spherical aberrations are larger, and the other off-axis aberrations are slightly smaller. The values of the six Zernike coefficients were, respectively, 0.9, 0.2, 0.2, 0.5, 0.5, and 1.0. Exit pupil plane wave aberration fitted with the above six coefficients $\varphi \left(x,y\right)=0.9Z1+0.2Z2+0.2Z3+0.5Z4+0.5Z5+Z6$. In addition, due to the use of a laser monochromatic light source, the amplitude of the light source is simulated with Gauss amplitude. The fitted phase difference distribution at the exit pupil and laser amplitude distribution are shown in Figure 4.

**Figure 5:**
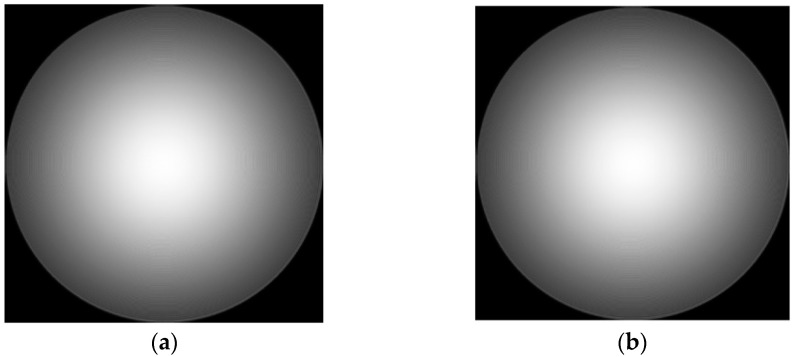
Focal plane and defocusing field distribution: (**a**) focal plane field distribution; and (**b**) optical field distribution of defocusing surface.

After fitting the aberration distribution at the exit pupil and the incident light source, the optical field can be transmitted according to the method described in Section 2, and the defocusing distance is 0.2 mm after the focus. Figure 5 shows the optical field distribution on the focal plane and defocusing plane.

**Figure 6:**
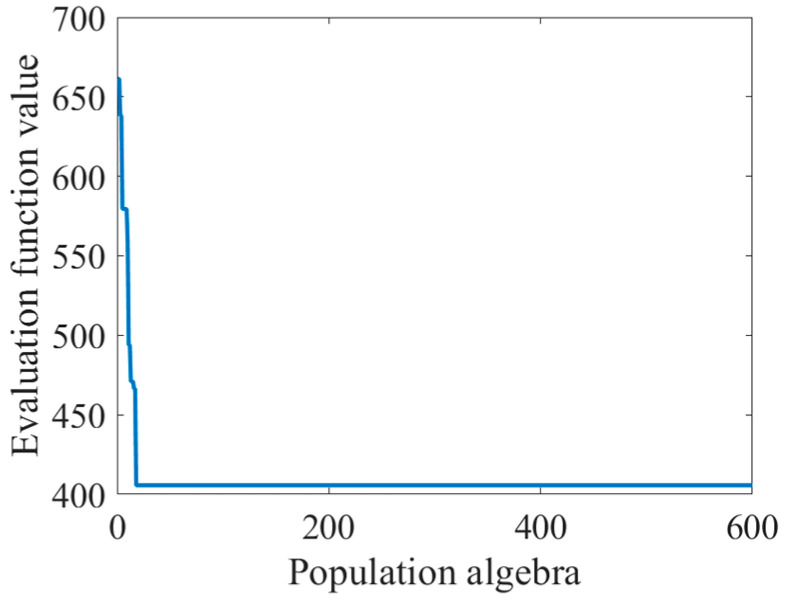
Graph of changes in evaluation function values as population algebra increases.

Substitute system parameters and the optical field data of the two planes into solution method of exit pupil frequency domain aberration, and the evaluation function of Zernike polynomial coefficients is obtained. The genetic algorithm was used to find the optimal solution to the evaluation function. The population size was set to 600, and the population algebra was set to 600 generations. The recovery of the Zernike coefficient and the change of evaluation function with population algebra are shown in Table 1 and Figure 6:

It can be seen from Figure 6 that the phenomenon of “precocity” is obvious. In the evolutionary process of the first few generations, the appearance of precocity individuals caused a rapid decline in the value of the evaluation function and, later, occupied a firm dominant position. Precocity is a common problem in the operation of the genetic algorithm. This phenomenon refers to the emergence of relatively excellent individuals in the early stage, and the offspring of the individual rapidly occupy an absolute proportion of the population after evolution. Precocity will lead to a decrease in the diversity of the population, thus, losing the ability to evolve.

**Figure 7:**
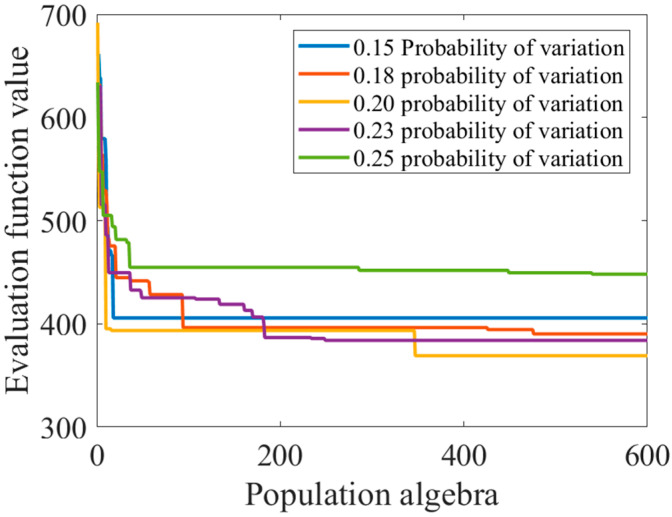
Graph of changes in evaluation function values as the population algebra increases.

The essence of prematurity is that the individuals in the group are homogenized seriously so that the high-order competition mode cannot be formed. The mutation stage can produce new individuals through gene mutation, increase the diversity of the population, have a certain probability of producing better individuals, and, to a certain extent, can inhibit the phenomenon of precocity puberty. In this section, multiple mutation probabilities are used to solve the problem. The solution results and population iteration process are as follows:

As can be seen from Table 2 and Figure 7, a mutation probability which is too small will reduce the probability of the emergence of new individuals, reduce species diversity, and cannot break the monopoly position of precocity individuals, but a mutation probability which is too large will destroy excellent genes, resulting in blind and meaningless evolution of the population. Although selecting the right mutation probability can obtain more excellent individuals to a certain extent, the “precocity” phenomenon is still very serious.

To restrain the “precocity” phenomenon in the optimization process of the genetic algorithm, some improvements are made in the three stages of the genetic algorithm selection, heredity, and variation.

(1) In the selection stage, dual competition is carried out based on random competition, and three copies of the population are made, which are population 1, population 2, and population 3, respectively. According to the mechanism of random competition, population 2 and population 3 compete with population 1, respectively, and half of the results of the two competitions are taken at equal intervals to form a new population. Dual competition reduces the risk of the loss of excellent genes caused by the competition between two excellent individuals to a certain extent and gives the second chance for the survival of the better genes;
(2) The crossover operator has a serious maturation effect on the search process. In order to restrain the prematurity and ensure the diversity of the population, in the cross-recombination stage, this paper carried out parallel cross-recombination based on the two-point crossover, that is, the parents were evenly divided into multiple groups, and each group exchanged genes in different regions, respectively. Concurrent recombination can effectively preserve the original genes of the parent while fully merging genes, which increases the diversity of the population;
(3) Non-uniform variation is adopted in the mutation stage. In order to protect the optimal individual from the influence of variation, the optimal individual is copied before the mutation operation. After the mutation is completed, an individual is randomly erased and the clone of the optimal individual of the previous generation is put into the new population. In addition, on this basis, the disaster model is introduced in this paper. When there is no new optimal individual in successive n generations, a natural disaster is introduced, that is, the mutation probability is slightly increased, and after m generations, if there is still no optimal individual, the mutation probability is increased again. From the above work, it can be seen that excessive mutation probability will hurt the evolution of the population, so the mutation probability cannot be increased uncontrollably, and an upper limit should be set. When a new optimal individual appears, the introduction of natural disasters is stopped and the initial mutation probability is returned.

**Figure 8:**
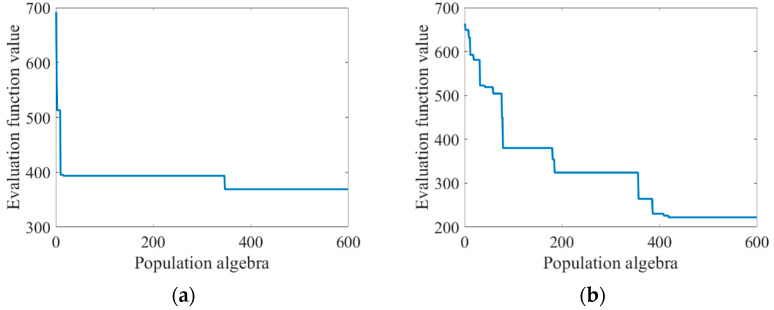
Graph of changes in evaluation function values as the population algebra increases: (**a**) before algorithm improvement; and (**b**) improved algorithm.

The improved genetic algorithm was used to optimize the Zernike coefficient, and the population size was still 600, and the population algebra was 600 generations. The optimal recovery before the improvement was compared, and the change curve of the recovery of the Zernike coefficient and the evaluation function along with the population algebra was shown in Table 3 and Figure 8.

As can be seen from Table 3, the improved genetic algorithm in this paper has a stronger optimization ability. It can be seen from Figure 8 that before the improvement of the algorithm, the “precocity” phenomenon was obvious. In the evolutionary process of the first few generations, the appearance of precocity individuals caused the value of the evaluation function to decline rapidly and occupy a dominant position later. After the improvement of the algorithm, the “precocity” phenomenon is suppressed. When population evolution stagnates, the introduction of the natural disaster model can accelerate population evolution to a certain extent.

**Figure 9:**
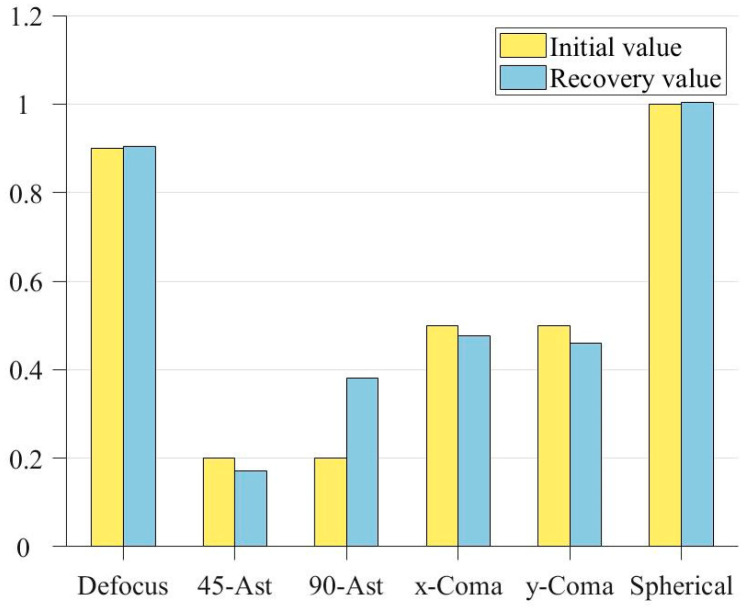
Bar comparison of Zernike coefficients.

**Figure 10:**
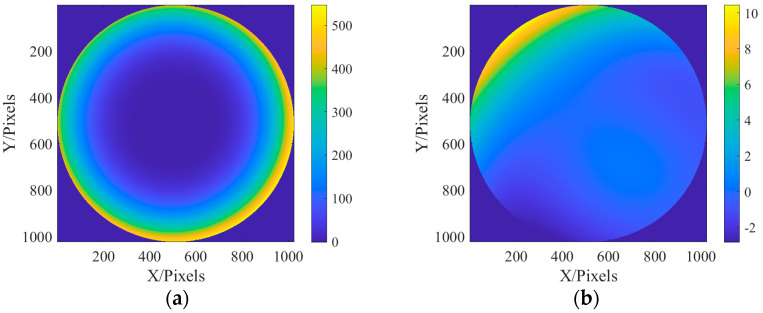
Aberration distribution and residual distribution of the recovered exit pupil: (**a**) recovered exit pupil aberration distribution; and (**b**) residual distribution.

In order to more intuitively show the recovery of the exit pupil phase by the improved genetic algorithm, a bar chart of coefficient comparison is drawn according to Table 3, as shown in Figure 9:

It can be seen from Figure 9 that the genetic algorithm has effectively recovered the approximate phase, and the recovery of the field of view defocus and first-order spherical aberration on the axis has been extremely close to the real value. Figure 10 shows the distribution of recovered exit pupil aberration and the residual distribution between it and the real one:

It can be seen from Figure 10 that the recovered phase distribution is in good consistency with the initial phase distribution set in Figure 4. It can be seen from the residual distribution that the residual is only 1% of the aberration. Generally speaking, the phase is recovered well.

**Figure 11:**
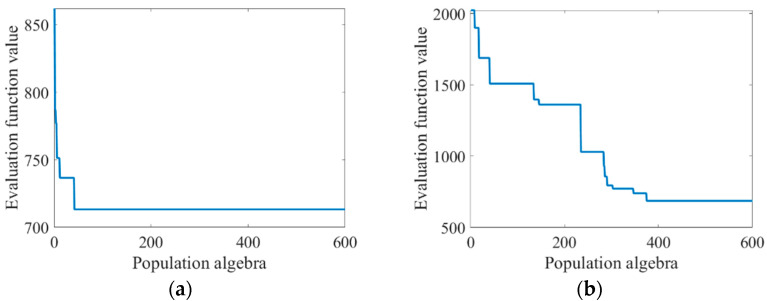
Graph of changes in evaluation function values as population algebra increases: (**a**) before algorithm improvement; and (**b**) improved algorithm.

The above work verifies the solving performance of the improved genetic algorithm with a small Zernike coefficient. When the Zernike coefficient is large, the distribution of the evaluation function will be more complex. The Zernike coefficients of the six aberrations were set as 5, 2, 2, 5, 5, and 9, which were solved by using the genetic algorithm before and after improvement. The population size was 600 and the population algebra was 600 generations. The recovery of Zernike coefficients and the iteration of the population were shown in Table 4 and Figure 11:

As can be seen from Table 4 and Figure 11, the solving ability of the classical genetic algorithm decreases to a large extent and the “precocity” phenomenon is serious after the increase of the Zernike coefficient. The improved genetic algorithm in this paper still maintains a relatively strong solving ability and obtains a high phase accuracy.

**Figure 12:**
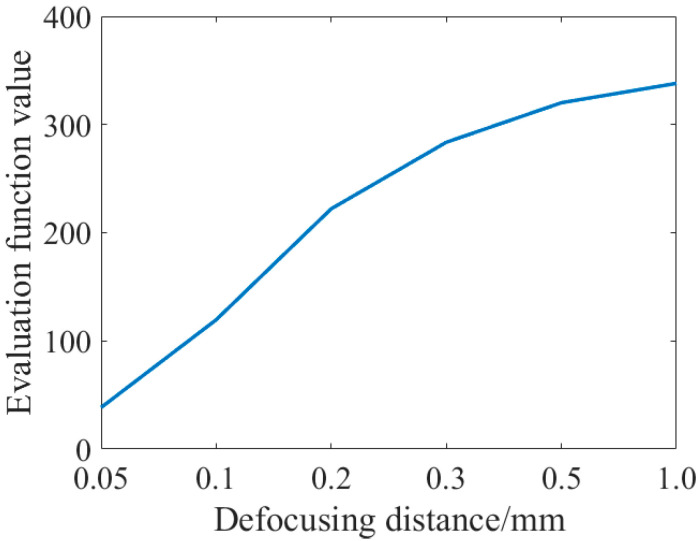
Evaluation function value and defocusing distance relationship graph.

**Figure 13:**
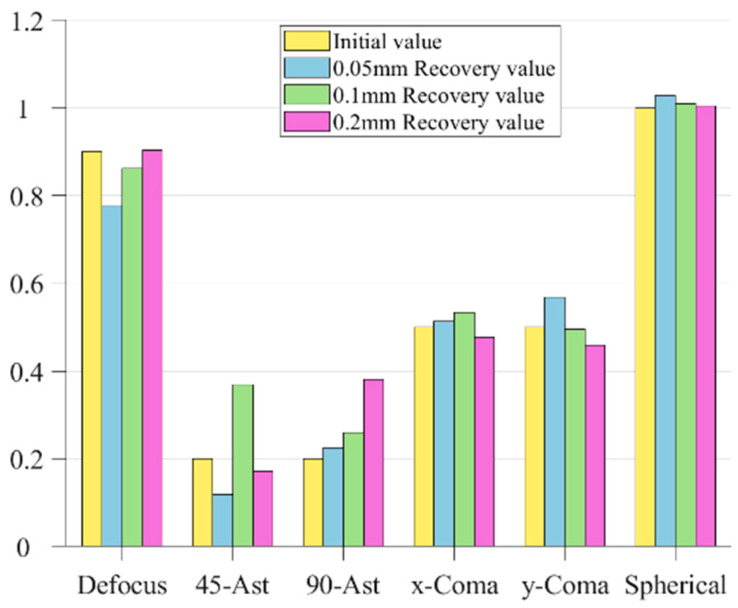
Comparison of phase recovery at different defocusing distances.

According to the solving principle of aberration in the exit pupil frequency domain in Section 2, the phase recovery algorithm mainly depends on the mutual iteration between the focal plane and a subsequent defocusing plane, so the defocusing distance will have a certain influence on the precision of phase recovery. Here, a simulation study is made on the influence of defocusing distance on the solving accuracy. In this section, the defocus values of 0.05 mm, 0.1 mm, 0.2 mm, 0.3 mm, 0.5 mm, and 1.0 mm were, respectively, selected for simulation using genetic algorithm. The recovery results of each Zernike coefficient were shown in Table 5. The changes in evaluation function values along with the defocus distance were shown in Figure 12:

As can be seen from the figure above, the value of the evaluation function increases with the increase of the defocusing distance, indicating that the shorter the defocusing distance, the better the evaluation of phase recovery. However, compared with the first three groups of data, as shown in Figure 13:

As can be seen from the figure above, although the value of the evaluation function decreases with the decrease of the defocusing distance, the ratio difference between the recovered Zernike coefficient and the fitted initial value phase increases somewhat, especially the two kinds of axial aberrations of defocusing and the first-order spherical aberration. The recovery of the defocusing of 0.05 mm is not as good as that of 0.1 mm and 0.2 mm.

It can be seen from the above work content that the precision of the phase recovery algorithm is not a simple linear relationship with the defocusing distance, and the smaller the defocusing distance is not the better. Too small a defocusing distance will lead to inaccurate phase information recovery, thus, affecting the imaging quality. The accuracy of the phase recovery algorithm is affected by a variety of factors, including the intensity of the light source, imaging depth, etc. When the defocusing distance is less than a certain value, the recovered phase information will appear as an “aliasing” phenomenon, namely, the blind area, as the depth information is blurred, resulting in reduced accuracy. Therefore, when choosing the defocusing distance, various factors such as the intensity of the light source, imaging depth, and optical path parameters should be comprehensively considered to find the appropriate defocusing distance.

## 4. Simulation Verification of the Frequency Domain Aberration Correction Model of a Coherent Imaging System

In this section, based on the theory in Section 2 and the research content in Section 3, the paraxial coherent imaging system is used to simulate and verify the feasibility of the frequency domain aberration correction model proposed in this paper.

**Figure 14:**
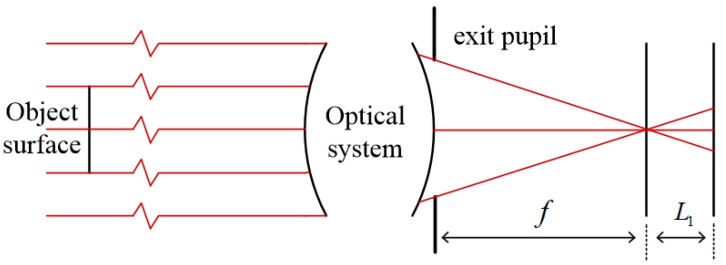
Schematic diagram of the light path in small-field diffraction imaging.

According to the above requirements, the exit pupil size of the final built optical system is 20 mm, the focal length is 250 mm, and the object distance is set to 7.5 m, which is 30 times the focal length. The light source still uses the monochromatic light source of 632.8 nm, and the amplitude distribution of the light source is simulated as Gauss distribution. Since angular spectral diffraction is used in the transmission process of the optical field, the size of the image-receiving surface is also set to 20 mm × 20 mm. According to the calculation, the radius of the Airy spot in the system is 9.65 μm, so the number of pixels on the receiving surface can be set to 2048 × 2048. The specific optical path diagram is shown in Figure 14.

**Figure 15:**
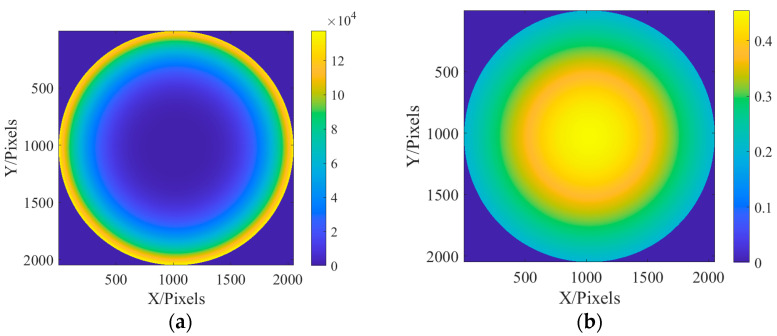
The distribution of exit pupil aberration and light source amplitude are fitted: (**a**) the fitted distribution of exit pupil aberrations; and (**b**) the amplitude distribution of the light source.

**Figure 16:**
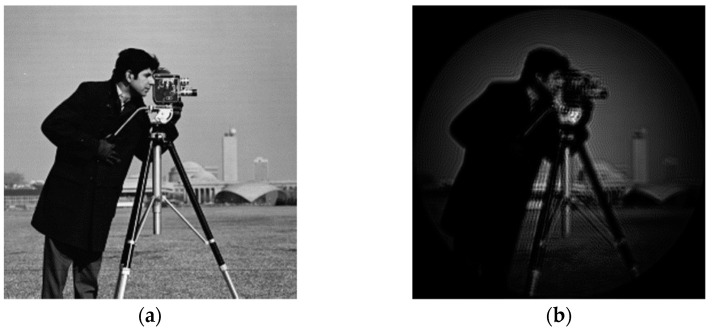
Cameraman original and Gauss image: (**a**) Cameraman picture original; and (**b**) Gauss image of optical system.

In this section, the classic Cameraman image is used for imaging. Since the pixel of Cameraman’s image is 512 × 512, it needs to be expanded to 2048 × 2048 pixels. In the simulation, the distribution of aberration at the exit pupil is still fitted by the Zernike polynomial, and six aberrations such as defocus (Z1), astigmatism (Z2, Z3) (45° and 0/90°), aberration (Z4, Z5) (x, y directions), and primary spherical aberration (Z6), are fitted. The coefficients of the Zernike polynomial are, respectively, 0.9, 0.2, 0.2, 0.5, 0.5, and 1.0. The fitted phase difference distribution at the exit pupil and amplitude distribution of the light source are shown in Figure 15:

A light source is used to irradiate Cameraman’s pictures, and Cameraman’s pictures are imaged. Figure 16 shows the Gauss image produced by Cameraman and the original image after the system:

As can be seen from the above figure, due to the diffraction effect of the coherent imaging system and the existence of the system aberration, the imaging quality is significantly reduced.

**Figure 17:**
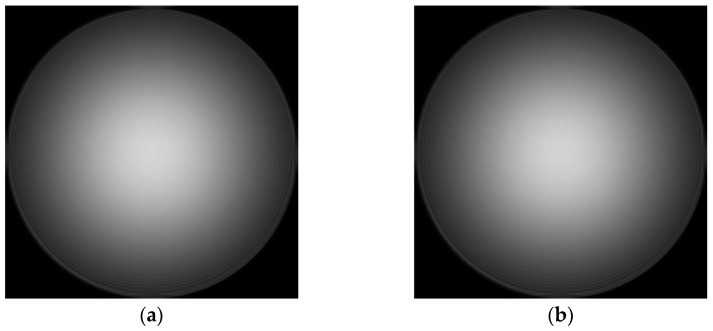
Optical field distribution in focal plane and defocusing plane: (**a**) focal plane field distribution; and (**b**) optical field distribution at 0.1 mm defocusing plane.

**Figure 18:**
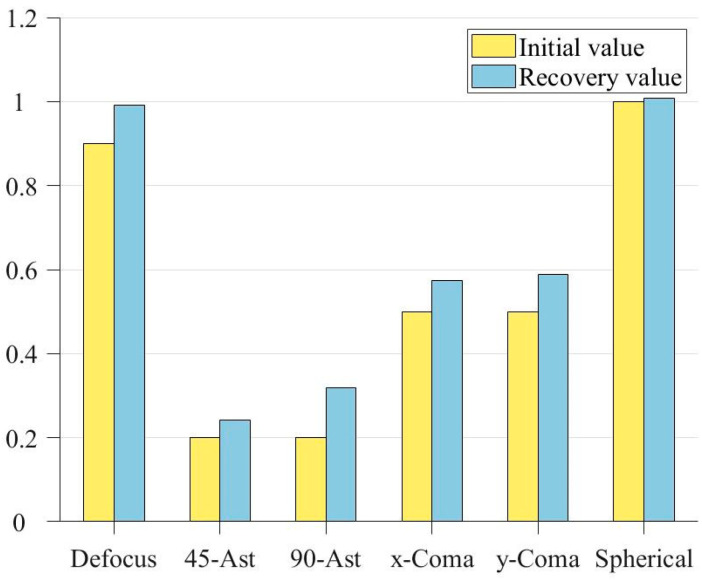
Bar comparison of Zernike coefficients.

In the process of solving the exit pupil phase, the improved genetic algorithm was used to optimize the evaluation function. The population size was set to 600, and the population algebra was 600 generations. According to the conclusions in Section 3, the defocusing distance of the defocusing plane is 0.1 mm, and the light intensity of the two planes collected is shown in Figure 17:

Table 6 shows the coefficients of the Zernike polynomial in frequency domain aberration of exit pupil recovered according to the above principle:

In order to directly show the recovery of each aberration, the square comparison diagram of coefficient distribution is drawn according to the above table, as shown in Figure 18.

**Figure 19:**
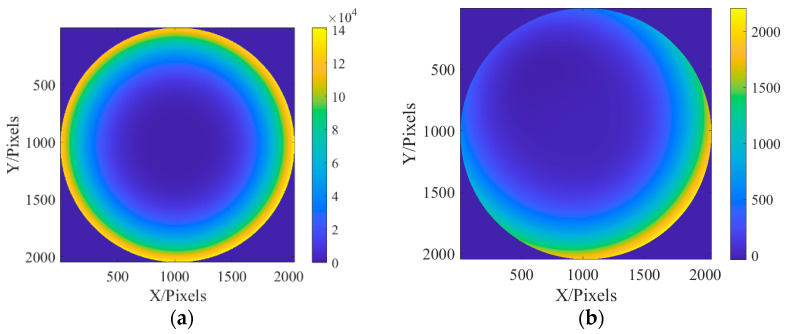
Aberration distribution and residual distribution of the recovered exit pupil: (**a**) recovered exit pupil aberration distribution; and (**b**) residual distribution.

**Figure 20:**
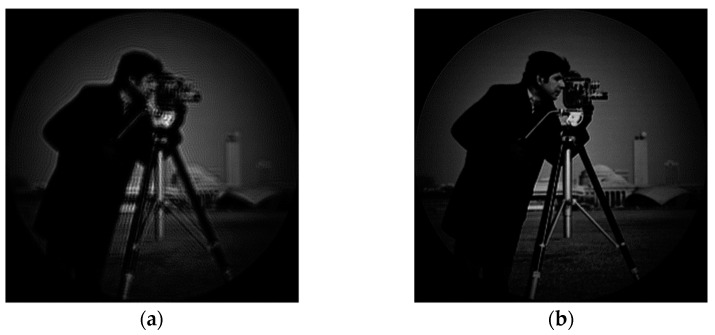
Gauss image before and after correction: (**a**) Gauss image of optical system; and (**b**) Gauss image after correction.

Figure 19 shows the recovered phase distribution and the residual distribution between the restored phase and the set exit pupil aberration:

The phase distribution of the aberration in the frequency domain at the exit pupil of the system is known as $H_{a}=e^{i\varphi }$, and the aberration correction filter $H_{ac}=e^{-i\varphi }$ of the system can be obtained by conjugate processing according to the aberration correction model in this paper. As can be seen from Equations (2)–(5), the filter can be used to directly correct the aberration of the system image in the frequency domain. Cameraman’s images before and after aberration correction are shown in Figure 20:

As can be seen from Figure 20, the image quality of the system has been significantly improved. Compared with the subject, the image quality of the background part with less information is significantly improved.

**Figure 21:**
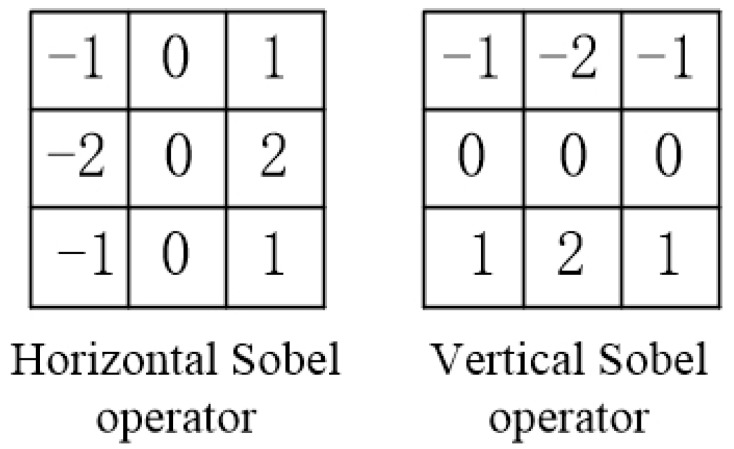
Sobel operator.

**Figure 22:**
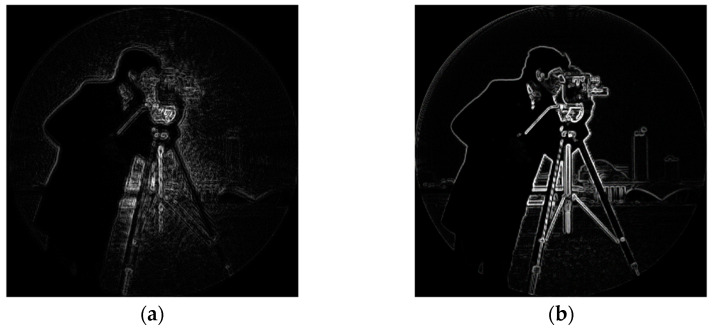
Comparison of edge information before and after Gauss image correction: (**a**) edge information of the original Gauss image; and (**b**) edge information of Gauss image after correction.

To characterize the image quality more quantitatively, the energy of gradient (EOG) was used to evaluate the image’s sharpness. EOG takes the square sum of the difference between the gray values of adjacent pixels in the x direction and the y direction as the gradient value of each pixel and adds the gradient values of all pixels as the evaluation value of picture clarity. Its expression is shown as follows:

(7) $$F=\sum_{x}\sum_{y}\left\{{\left[I\left(x+1,y\right)-I\left(x,y\right)\right]}^{2}+{\left[I\left(x,y+1\right)-I\left(x,y\right)\right]}^{2}\right\},$$

where $I\left(x,y\right)$ is the gray value of the image at the pixel $\left(x,y\right)$. The sharpness evaluation results obtained by using the energy of the gradient are shown in the Table 7:

As can be seen from the above table, after correction, the energy gradient of the image goes up from 3.54519 × 10^10^ to 4.05023 × 10^10^, increasing by 14.25%. In order to show the clarity of the image more directly, the edge information of the image is extracted. The spatial filtering method is used to obtain the gray change of each pixel in its neighborhood and the gradient change inside the image to obtain the edge information of the image [17,18]. In this paper, the first-order Sobel operator is used to extracting image edge information, which can significantly suppress the influence of noise, as shown in Figure 21:

The Sobel operator divides into two directions, calculates the gradient components Gx and Gy in both directions, compresses them to the interval [0, 255], and combines the gradient to obtain $G\left(x,y\right)$. Set the appropriate threshold so that the output is 1 if it is greater than the threshold and zero if it is less than the threshold. The image generated by the system and the edge information extraction results after correction are shown in Figure 22.

As can be seen from the above figure, the image corrected by aberration has richer edge information and sharper edge, and the edge information is improved particularly significantly at the straight line and smooth curve of the background, which verifies the feasibility of the image quality improvement method of the coherent imaging system proposed in this paper.

## 5. Experimental Verification of the Frequency Domain Aberration Correction Model for a Coherent Imaging System

The above work verifies the feasibility of the image quality enhancement method of the coherent imaging system proposed in this paper through simulation studies. In this section, a specific experimental optical path is built to verify the aberration correction model.

**Figure 23:**
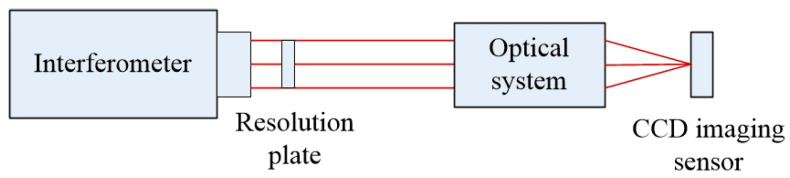
Schematic diagram of experimental optical path.

**Figure 24:**
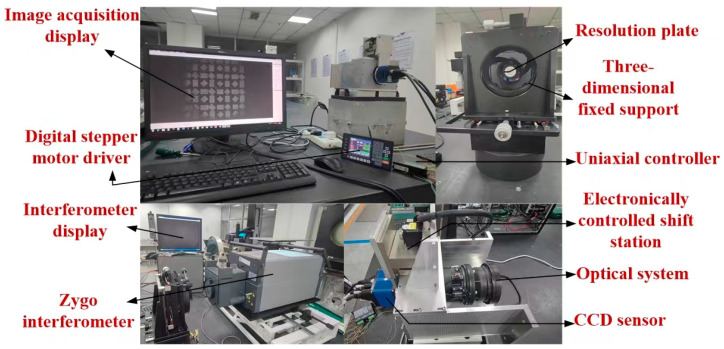
Layout of experimental optical path device.

According to the theoretical model in this paper, establish a paraxial imaging system, and the experimental diagram is shown in Figure 23:

The actual experimental optical path is shown in Figure 24. Since the experimental optical path is long, it is shown by the module in the figure.

**Figure 25:**
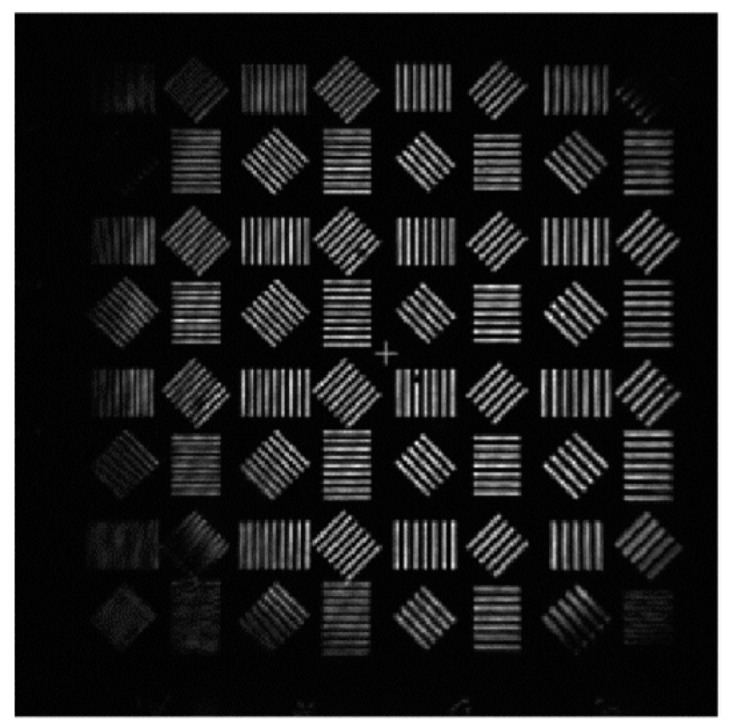
Gauss image light intensity distribution.

**Figure 26:**
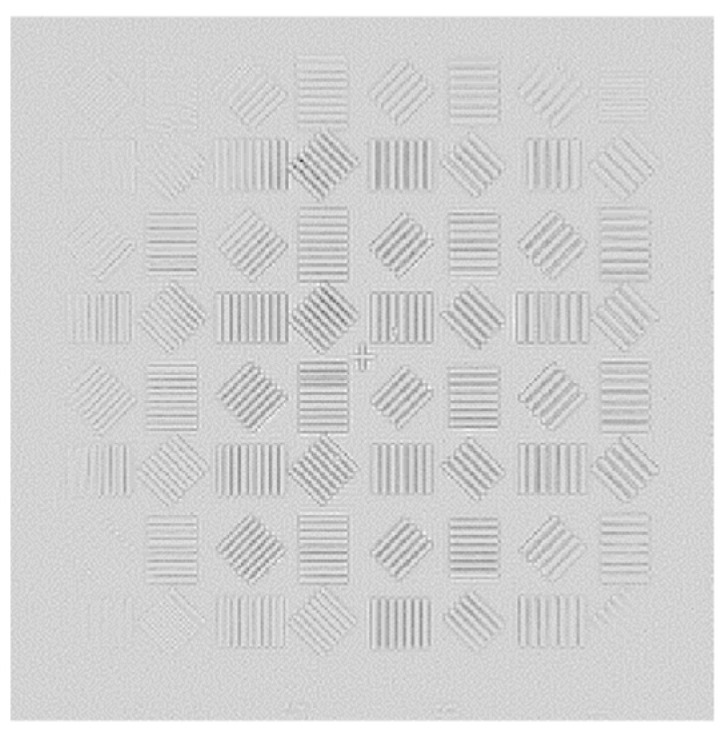
Phase distribution recovered by the accelerated angular spectrum iteration method.

**Figure 27:**
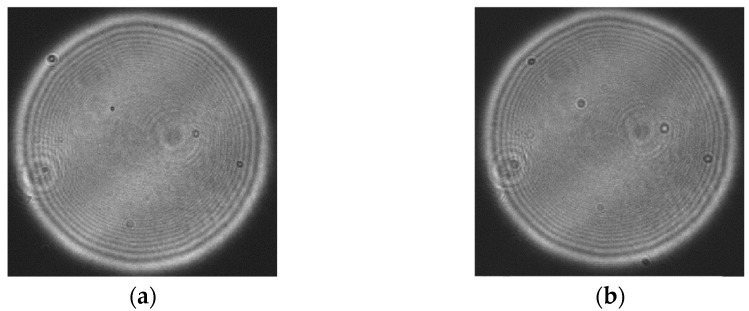
Optical field distribution in focal plane and defocusing plane: (**a**) focal plane intensity distribution; and (**b**) optical field distribution at 0.1 mm defocusing plane.

The light source is a 632.8 nm monochromatic light source from the Zygo interferometer. The focal length of the optical system used is 149.98 mm and the exit pupil size is 32.22 mm. A panchromatic CCD image sensor with a pixel size of 7.4 μm×7.4 μm and resolution of 4864×3232 is placed behind the optical system. In this paper, 2048×2048 sampling points are intercepted in the phase recovery algorithm. The collected Gaussian image is shown in Figure 25:

In the experiment, it is verified that the time field information is collected by the CCD sensor, which can only obtain the light intensity information of the optical field, but not the phase information. Therefore, it is necessary to restore the phase distribution of the Gaussian image to obtain the complete optical field distribution. In this paper, the accelerated angular spectral iteration method is used to recover the phase of the Gauss image [19], and the recovered phase picture is shown in Figure 26:

After obtaining the phase distribution of the Gaussian image, the exit pupil aberration should be solved so that the aberration filter can be constructed to correct the Gauss image. According to the aberration correction model in this paper, the optical intensity distribution of the focal plane and the defocusing plane should be collected. In this process, optical path parameters should be strictly consistent with the Gauss image acquisition optical path, and only the resolution plate should be removed so that the imaging system can directly image the interferometer optical source. In this paper, the defocusing distance is 0.1 mm, and the optical intensity distribution of the obtained focal plane and the defocusing plane is shown in Figure 27.

**Figure 28:**
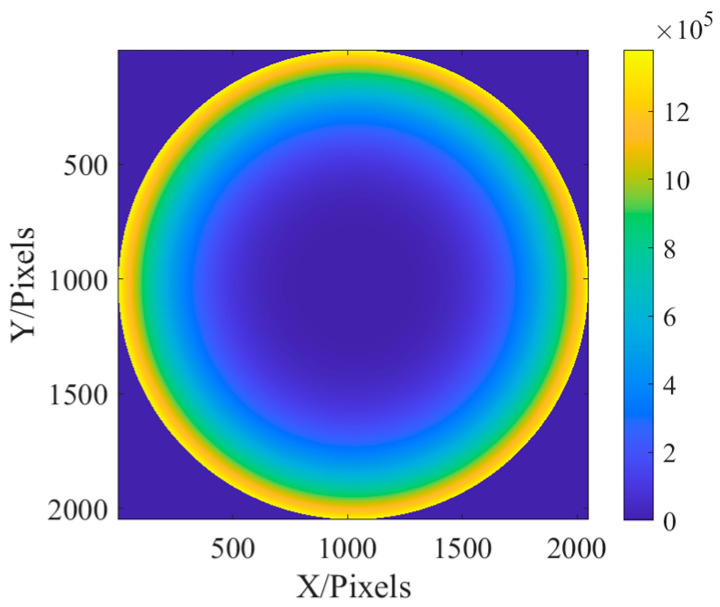
Recovery phase diagram.

Six primary aberrations, such as defocus (Z1), astigmatism (Z2, Z3) (45 degrees and 0/90 degrees), coma (Z4, Z5) (x and y directions), and primary spherical aberration (Z6), are still used to solve the aberration in the frequency domain of exit pupil. An improved genetic algorithm is used to optimize the evaluation function. The distribution of aberration in the frequency domain of exit pupil recovered is shown in Figure 28.

**Figure 29:**
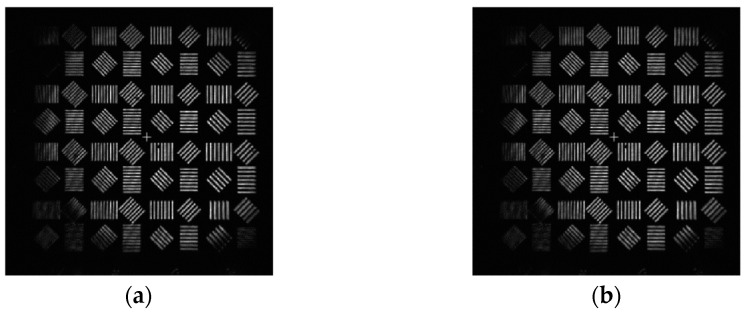
Gauss image and corrected image produced by the system: (**a**) Gaussian image of the system; and (**b**) the corrected image.

The phase distribution of aberration at the exit pupil is known, and the aberration correction filter for the imaging system can be obtained by conjugate processing it, and, then, the aberration correction of the Gauss image in the frequency domain is conducted. The correction results are as Figure 29:

The energy of gradient (EOG) is used to evaluate the imaging quality, and the results are shown in Table 8:

As can be seen from Table 8, after filtering and correction, the energy gradient function value of the system is increased from 58,550,562 to 59,858,468, an increase of 2.23%, which proves that the image sharpness is improved and verifies the feasibility of the proposed algorithm.

## 6. Conclusions

This paper presents a method to correct the aberration of a coherent imaging optical system, which does not need to change the original optical path design and takes into account the imaging quality and miniaturization requirements of the imaging system. Firstly, the frequency domain aberration at the exit pupil is solved based on the idea of phase recovery, and the genetic algorithm is used to optimize the Zernike coefficient, which greatly improves the solving accuracy through the improvement in this paper. After solving the aberration distribution in the frequency domain of the exit pupil, a conjugate aberration filter is constructed to filter the image in the frequency domain. The image generated by the system is used as the intermediate image to directly output the corrected image. Finally, the paraxial imaging optical path is simulated and verified by experiments. The aberration correction method proposed in this paper is used to improve imaging quality. The results show that the energy gradient function value of the simulated image is increased by 14.25% after aberration correction, and the experimental results are improved by 2.23%, which verifies the feasibility of the aberration correction method of the coherent imaging system proposed in this paper. In the following work, the application of this aberration correction method in the imaging optical path of the large field of view can be further studied to expand the application range.

## Figures and Tables

**Table 1:** The recovery of the Zernike coefficient.

	Defocus	45 Astigmatic	90 Astigmatic	X Coma	Y Coma	Spherical Aberration
Initial value	0.9	0.2	0.2	0.5	0.5	1
Recovery value	0.7450	0.2421	0.0201	0.5838	0.4162	1.0473

**Table 2:** The recovery results of the Zernike coefficient were obtained using different probability of variation.

	Defocus	45 Astigmatic	90 Astigmatic	X Coma	Y Coma	Spherical Aberration	Appraise Value
Initial value	0.9	0.2	0.2	0.5	0.5	1	---
0.15 recovery value	0.7450	0.2421	0.0201	0.5838	0.4162	1.0473	405.6673
0.18 recovery value	0.7681	0.0804	0.4365	0.6709	0.3910	0.9571	390.2093
0.20 recovery value	0.9913	0.5460	0.3194	0.6910	0.5221	1.0404	369.0254
0.23 recovery value	0.8624	0.3916	0.1338	0.4747	0.6585	0.9573	383.9376
0.25 recovery value	1.0729	0.5126	0.5986	0.7880	0.6462	1.0733	448.1115

**Table 3:** The recovery of the Zernike coefficient.

	Defocus	45 Astigmatic	90 Astigmatic	X Coma	Y Coma	Spherical Aberration	Appraise Value
Initial value	0.9	0.2	0.2	0.5	0.5	1	
Initial recovery value	0.9913	0.5460	0.3194	0.6901	0.5221	1.0404	369.0254
Improved recovery value	0.9094	0.1721	0.3807	0.4770	0.4594	1.0048	222.1505

**Table 4:** The recovery of the Zernike coefficient.

	Defocus	45 Astigmatic	90 Astigmatic	X Coma	Y Coma	Spherical Aberration	Appraise Value
Initial value	5	2	2	5	5	10	
Initial recovery value	4.0217	3.6801	2.3041	4.7981	5.1766	9.1937	713.2161
Improved recovery value	4.7557	2.0356	2.0176	5.2580	5.2164	8.8934	686.7341

**Table 5:** Relationship between recovery of Zernike coefficient and defocusing distance.

	Defocus	45 Astigmatic	90 Astigmatic	X Coma	Y Coma	Spherical Aberration	Appraise Value
Initial value	0.9	0.2	0.2	0.5	0.5	1	---
0.05 mm Recovery value	0.7777	0.1194	0.2244	0.5143	0.5681	1.0278	38.4331
0.1 mm Recovery value	0.8624	0.3685	0.2603	0.5334	0.4949	1.0098	119.5324
0.2 mm Recovery value	0.9040	0.1721	0.3807	0.4770	0.4594	1.0048	222.1505
0.3 mm Recovery value	0.9865	0.3755	0.1611	0.5062	0.5616	0.9826	283.6212
0.5 mm Recovery value	0.8956	0.1892	0.4135	0.6316	0.4280	0.6565	320.1431
1.0 mm Recovery value	0.9040	0.0519	0.1348	0.5441	0.3606	1.0390	338.0757

**Table 6:** The recovery of the Zernike coefficient.

	Defocus	45 Astigmatic	90 Astigmatic	X Coma	Y Coma	Spherical Aberration
Initial value	0.9	0.2	0.2	0.5	0.5	1
Recovery value	0.9920	0.2419	0.3184	0.5750	0.5888	1.0088

**Table 7:** Gauss image energy of gradient before and after correction.

	Energy of Gradient Value
Gauss image of the system	3.54519 × 10^10^
Gauss image after correction	4.05023 × 10^10^

**Table 8:** Gauss image energy of gradient before and after correction.

	Energy of Gradient Value
Gauss image of the system	58,550,562
Gauss image after correction	59,858,468

## Data Availability

Data underlying the results presented in this paper are not publicly available at this time but maybe obtained from the authors upon reasonable request.
